# Opportunities and lessons learned from a retrospective analysis of administrative billing data to understand the language profile of high-risk close contacts of COVID-19 cases in Ontario

**DOI:** 10.14745/ccdr.v50i10a06

**Published:** 2024-10-03

**Authors:** Andrea Chambers, Mark A Cachia, Jessica P Hopkins

**Affiliations:** 1 Public Health Ontario, Toronto, ON; 2 Department of Health Research Methods, Evidence, and Impact, McMaster University, Hamilton, ON

**Keywords:** contact tracing, COVID-19, SARS-CoV-2, language concordance, pandemic

## Abstract

**Background:**

During a public health emergency, it is vital to have access to data sources that can identify communities disproportionately affected and to ensure public health communications are meeting the needs of diverse populations.

**Objective:**

To explore how administrative billing data for language interpretation services could be used as an additional source of information to understand the language profile of high-risk close contacts of COVID-19 cases.

**Methods:**

A retrospective descriptive analysis was conducted using administrative billing data from Public Health Ontario's Contact Tracing Initiative from May 2020 to February 2022. Data from the Contact Tracing Initiative were utilized to identify drivers that could have influenced patterns in language interpretation requests. Trends were compared with community language profiles using 2021 Canadian Census data.

**Results:**

Interpreters responded to 2,604 requests across 38,518 interpretation minutes and provided information in 50 different languages. The top five requested languages were French, Arabic, Spanish, Punjabi and Mandarin. Five distinct periods were identified of different language predominance including Spanish in spring/summer 2020, French in summer/fall 2020 and Arabic in spring 2021. Overall, these trends aligned with the language profile of health units contributing most submissions.

**Conclusion:**

Public health agencies could benefit from using existing secondary data sources to understand the language interpretation needs of their communities. This study also demonstrated how existing data sources could be used to help assess how communities are being disproportionately affected by public health emergencies and how this might change over time.

## Introduction

The use of case and contact management is a foundational public health approach to control the spread of infectious diseases. Conducting case and contact management was an important priority for many jurisdictions during the early phases of the COVID-19 pandemic (1). Forward contact tracing involves cases identifying people (“contacts”) who may have been exposed to the SARS-CoV-2 during their period of communicability. Public health agencies then communicate with high-risk contacts to advise them of the exposure and provide information on testing, isolation requirements and enabling supports. For the delivery of case and contact management to be effective and equitable, information and support needs to be delivered in a community’s preferred language (2–4).

Socioeconomic data collected early during the COVID-19 pandemic in Ontario helped describe how some communities were disproportionately impacted and early results emphasized the importance of looking at language ability (5,6). In Ontario, approximately 16% of the population predominantly speaks a non-official language at home (7). An analysis of patterns of testing and test results early during the pandemic found that lack of English or French language ability was associated with lower testing but higher percent positivity among recent adult immigrants and refugees in Ontario (6).

Collection of individual-level socioeconomic data from COVID-19 cases in Ontario was not extended to collection of information from high-risk close contacts of COVID-19 cases. We see this as a gap as the disproportionate impacts of the pandemic extend to other outcomes and experiences, including the mental health and financial impacts of multiple and prolonged periods of isolation associated with being identified as a high-risk close contact (8). Moreover, primary data collection efforts were time-intensive and several factors impacted data completeness, accuracy and sustainability (9).

Given the gaps in understanding how language interpretation services have been utilized among high-risk contacts during the COVID-19 pandemic, this study aimed to 1) outline the steps used to leverage secondary data sources to understand the language profile of high-risk close contacts and 2) describe how this type of analysis can help investigate disproportionate impacts of the COVID-19 pandemic.

## Methods

### Setting

This study leveraged data from Public Health Ontario’s COVID-19 Contact Tracing Initiative (CTI). Between April 2020 and February 2022, Ontario’s 34 local public health units (PHUs) could use the CTI to help manage the volume of work associated with contact notification.

Provincial and federal government agencies provided support for initial and follow-up phone calls to high-risk close contacts of confirmed or probable cases of COVID-19 (10). If a contact required interpretation services or requested if services were available, the interviewer would dial the interpretation service provider to provide simultaneous interpretation in the contact’s preferred language. How this program was developed and used by local PHUs in Ontario has been described in more detail in a separate publication (10).

Below, we have outlined the four-step process used to conduct a descriptive retrospective analysis of secondary data sources and a visual analysis of trends to describe the language profile of high-risk contacts.

### Step 1: Analyze language interpretation services data

We obtained administrative billing data from the interpretation services vendor from May 4, 2020 (first billing date), to February 25, 2022 (last day of operations and possible billing date). The billing data included a line listing reflecting interpretation requests, the language requested and the call duration with no missing data across the variables of interest. We computed the frequency of encounters with language interpretation services, the cumulative total interpretation time in minutes and the median interpretation time and interquartile range (IQR) for each language and overall. A visual analysis of time trends was used to identify shifts in language predominance.

### Step 2: Identify drivers that could be influencing patterns in language interpretation requests

Data from the CTI were utilized to examine trends over time in the volume of high-risk contacts, independent of translation requests, submitted to the program. We described changes over time in which health units were submitting the majority of contacts to the CTI.

### Step 3: Compare trends with region-specific census data

For comparison purposes, data from the 2021 Canadian Census were extracted to summarize information about the primary languages spoken most often at home for Ontario and regions supported by Ontario’s 34 PHUs (11). Specifically, we focused on the number of single responses (i.e., the number of people who gave only one language) for the language spoken most often at home.

### Step 4: Identify patterns and discrepancies

After completing steps 1 to 3, comparisons were made across data sources. Two primary questions helped identify patterns:

• Do the top languages requested for interpretation align with the language profiles (according to the 2021 Census) for regions contributing the most submissions to the program?

• Do changes in the top languages requested over time align with changes in which local PHUs were submitting a high volume of contacts?

## Results

There were 972,625 calls to high-risk contacts over 21 months (May 14, 2020, to February 7, 2022) with fewer than 1% of calls requiring language interpretation support. Interpreters responded to 2,604 requests, totaling 38,518 interpretation minutes (**Table 1**). Overall, there were 50 different languages requested (Table 1). For the entire observation period, the top five languages were French, Arabic, Spanish, Punjabi and Mandarin, accounting for 69.2% of all interpretation minutes. Among the top five languages requested, median interpretation time varied from 7.0 minutes (French) to 13.5 minutes (Arabic), with IQRs ranging from a low of 4.0 minutes (French) to a high of 25.0 minutes (Arabic).

**Table 1:** Requests for language interpretation support during phone calls with high-risk contacts of COVID-19 cases supported by Public Health Ontario’s Contact Tracing Initiative, May 14, 2020, to February 17, 2022

Language	Number of encounters	Cumulative minutes	Median (IQR)
Akan	2	15	7.5 (6.8–8.3)
Albanian	14	222	8.0 (4.3–16.8)
Amharic	3	43	16.0 (9.5–20.0)
Arabic	370	6,642	13.5 (7.0–25.0)
Bengali	3	49	20.0 (10.5–24.0)
Cantonese	73	1,166	13.0 (5.0–23.0)
Croatian	4	78	22.0 (12.3–29.3)
Czech	2	43	21.5 (20.8–22.3)
Dari	11	213	19.0 (11.0–23.0)
Estonian	1	5	N/A
Farsi	41	528	8.0 (5.0–17.0)
French	803	9,203	7.0 (4.0–16.0)
German	30	175	6.0 (4.0–7.8)
Greek	4	80	20.5 (9.3–31.3)
Gujarati	2	8	N/A
Hindi	53	768	10.0 (4.0–20.0)
Hungarian	9	132	7.0 (5.0–15.0)
Indonesian	2	54	27.0 (22.5–31.5)
Italian	27	322	10.0 (6.0–15.5)
Japanese	3	21	5.0 (5.0–8.0)
Karen	2	53	26.5 (19.3–33.8)
Khmer	1	18	N/A
Korean	24	456	15.5 (6.3–31.3)
Laotian	2	13	6.5 (4.8–8.3)
Mandarin	133	2,146	9.0 (5.0–20.0)
Nepali	5	90	18.0 (15.0–21.0)
Pashto	1	11	N/A
Polish	25	497	20.0 (10.0–24.0)
Portuguese	51	968	16.0 (7.5–25.5)
Punjabi	221	3,657	10.0 (5.0–22.0)
Rohingya	3	15	2 (1.5–7.0)
Romanian	1	69	N/A
Russian	10	167	14 (5.0–19.5)
Serbian	12	221	15 (11.3–25.0)
Shanghainese	1	10	N/A
Somali	27	379	9.0 (5.0–22.0)
Sorani	3	14	5 (4.0–5.5)
Spanish	316	5,009	13 (5.0–23.0)
Sudanese Arabic	2	53	26.5 (18.3–34.8)
Swahili	5	35	4.0 (4.0–12.0)
Tagalog	11	165	6.0 (5.0–24.0)
Taiwanese	1	5	N/A
Tamil	62	915	9.0 (5.0–23.0)
Telugu	2	9	4.5 (4.3–4.8)
Thai	16	409	17.0 (13.8–24.5)
Tigrigna/Tigrinya	42	810	12.5 (7.0–25.8)
Turkish	14	168	12.5 (5.3–14.8)
Ukrainian	10	163	16.0 (11.8–18.3)
Urdu	38	434	7.0 (4.0–14.8)
Vietnamese	106	1792	12.5 (4.0–23.0)
Total	2,604	38,518	10.0 (5.0–20.0)

Abbreviations: IQR, interquartile range; N/A, not applicable

**Figure 1:**
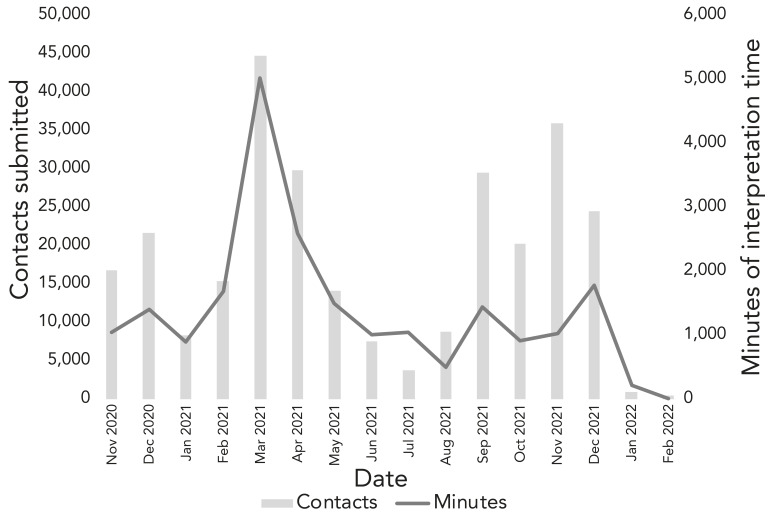
High risk-contacts submitted to Public Health Ontario’s COVID-19 Contact Tracing Initiative and language interpretation requests (in minutes), November 2020 to December 2021

We noted that overall time trends for language interpretation minutes aligned with trends in the volume of contacts submitted to the CTI, with some exceptions (**Figure 1**). For example, there were periods where the number of interpretation minutes was high relative to contacts submitted, including the period from January to July 2021.

We examined time trends to assess how the top requested languages for interpretation services changed over the observation period. Four periods of interest were identified to investigate further.

**Figure 2:**
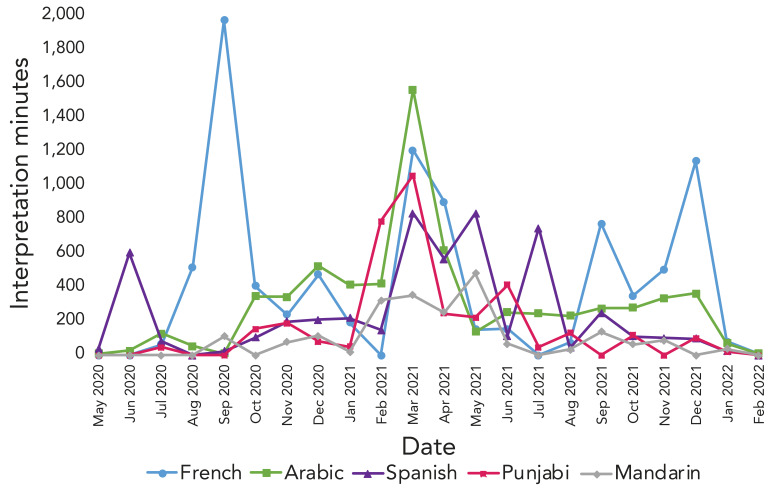
Language interpretation minutes for the top five requested languages over the study period

**Observation 1:** In September 2020, there was a rise in French language interpretation requests (**Figure 2**). The following local PHUs submitted approximately 94% of the contacts to the CTI during this month (number of contacts): Ottawa (n=1,204), Halton (n=710), Durham (n=666), York (n=509) and Niagara (n=346) (Table 1) (supplemental data available from the corresponding author). According to the 2021 Canadian Census, French was the most common non-English language spoken most often at home in Ottawa, accounting for approximately 40% of all non-English languages reported (supplemental data available from the corresponding author).

**Observation 2:** Arabic was the most common language requested for interpretation between November 2020 and March 2021 (Figure 2). The following PHUs submitted approximately 60% of the contacts in March 2021 (number of contacts): Durham (n=6,325), Peel (n=5,804), Sudbury & Districts (n=4,890), Halton (n=4,059), Hamilton (n=2,657) and Niagara (n=2,452) (supplemental data available from the corresponding author). Peel had the highest percentage of individuals reporting a non-official language spoken at home (33%) according to the 2021 Census (supplemental data available from the corresponding author). Punjabi was the most common language spoken at home in this region (32%), with Arabic coming in third (5.3%) (supplemental data available from the corresponding author).

**Observation 3:** In the early spring of 2020 (May 2020) and between March and July 2021, there was a rise in language interpretation requests for Spanish (Figure 2), with this language becoming predominant in the spring-summer period when Waterloo, Peel, Halton and Grey Bruce PHUs continued to submit high volumes of contacts (supplemental data available from the corresponding author). Halton and Waterloo regions also submitted high volumes of contacts in May 2020 (supplemental data available from the corresponding author). We noted that Spanish was not among the top three non-official languages spoken most often at home in these regions according to the 2021 Census.

**Observation 4:** In the final months of the program (September 2021 to December 2021), French became the predominant language requested for interpretation (Figure 2). This could be attributed to the sudden rise in submissions from Eastern Ontario and Sudbury & Districts (supplemental data available from the corresponding author). These PHUs have a large proportion of the population that speaks French most often at home (supplemental data available from the corresponding author). Public health units that submitted a high volume of contacts during these four months included (number of contacts): Durham (n=18,146), Waterloo (n=15,150), Niagara (n=12,363), Sudbury & Districts (n=8,236), Peel (n=6,437) and Eastern Ontario (n=6,159) (supplemental data available from the corresponding author).

## Discussion

Interpreters provided over 38,500 minutes of interpretation services in 50 languages for the CTI. Some of the shifts in language predominance could be explained by changes in which local PHUs were submitting a high volume of contacts to the CTI and the associated language profiles of those communities.

There were two periods when the patterns in language interpretation requests could not be explained by examining which health units were driving submissions and their community’s language profiles. The predominance of Arabic interpretation requests is an interesting finding that could represent the disproportionate impact of COVID-19 pandemic on Arabic-speaking communities. This observation is consistent with the findings of an analysis of race-based data collected by Ontario PHUs, where Middle Eastern communities experienced disproportionality high crude per capita rates of COVID-19 infection (9).

The increase in Spanish interpretation requests is another interesting finding as Spanish is not among the top three non-official languages spoken most often at home for the health unit regions that were driving submissions. This observation is consistent with the findings of the analysis of race-based data collected by Ontario PHUs, where Latino communities in Ontario experienced the highest crude per capita rates of COVID-19 infection in Ontario (9). In the absence of systematic collection of race-based data, lack of concordance between language interpretation requests and a community’s language profile could prompt further investigation to identify potential disproportionate impacts of disease that should be addressed.

Our work shows there is extreme variability between the average lengths of interpretation encounters. Interpretation is more than a direct translation. There is a need to incorporate cultural contexts and unique characteristics of the target language into scripts that were written in English. We believe this this is an important area for future study with opportunities to continue to build on work that aims to improve technology and training for effective communication that is mediated by an interpreter.

### Strengths and limitations

Key strengths of this study were the novel use of administrative data for understanding public health communication needs and completeness of the data set spanning the full program duration. There are important limitations and caveats to the data available for this exploratory analysis that we made note of. Individual encounters could have involved calls to households with one or more persons or a proxy (e.g., parent for a child); therefore, we were unable to identify the number of unique contacts.

This was also an exploratory descriptive study with limitations in being able to control for potential confounding factors. The contacts supported by the CTI are a subset of high-risk contacts in Ontario that were submitted by PHUs based on program criteria, which changed over time in response to PHU needs and provincial policy directions. There will be a less accurate picture of interpretation needs during periods when there was a high volume of COVID-19 cases when some case and contact management activities were modified to prioritize other COVID-19 response activities.

The use of administrative billing data for requests for interpretation services from an external vendor may not fully capture all language interpretation needs. The need for interpretation services may not have always been requested by the contact or recognized by the interviewer. The effectiveness of training on accessing interpretation services and the consistency in which these services were recommended by interviewers was not assessed. It is important to further understand barriers to effective communication and other factors, including cultural preferences, to continue to improve language services and the overall delivery of public health information.

## Conclusion

Public health agencies could benefit from using existing secondary data sources to understand the language interpretation needs of their communities. This study also demonstrated how existing data sources could be used to help assess how communities are being disproportionately affected by public health emergencies.
